# Gender regimes in health systems

**DOI:** 10.3402/gha.v7.26696

**Published:** 2014-12-09

**Authors:** Carlos Alvarez-Dardet

**Affiliations:** Public Health and Preventive Medicine, Public Health Research Group, The University of Alicante, Alicante, Spain

In short, what Elisa Chilet has found in her recent thesis dissertation entitled ‘Gender bias in clinical research, pharmaceutical marketing and the prescription of drugs’, a review of which is published in this issue of the journal (1), is a significant amount of gender bias. Elisa found the health system to be pervaded by a construct of discrimination against women in virtually all areas of the health system she examined, both in the private sector (pharmaceutical companies) and in the Spanish national health service, from the production of knowledge using clinical trials to marketing practices on the web and above all medical practice in prescription of extremely common drugs for common conditions. She produced fine papers published in good journals and now this recompilation. Her intellectual travel during her PhD training is worthwhile; coming from a biology background, Elisa Chilet arrived to public health and gender studies under the supervision of Maria Teresa Ruiz Cantero, showing how flexibility and adaptation to new challenges is an important asset in health research.

Her positioning in feminist empiricism seems adequate to produce information of better scientific quality, free of gender bias. However, perhaps the problem is not just bad science due to methodological errors, but a science constructed as biased against women. After signaling this, the actions required to ameliorate the problem are not obvious; just producing better information is not enough to change the situation of discrimination against women.

The crucial question in my view after reading her paper is ‘so what?’; she has produced new knowledge, had relevant papers in good-quality journals, maybe meriting attention and quotations in the coming years, but the translation into action of the produced knowledge should be refined; to solve this, in my opinion, would obviously require taking power into account.

These processes cannot be understood without taking into account what have been called gender orders and gender regimes by Connell (2). Gender order is the social construction of the power relations of genders, which is reflected in institutions (such as the welfare state institutions), values, and directions in male-dominated gender regimes (3). After Elisa's papers, I feel we are faced with true gender regimes in health systems. Gender regimes should be actively deconstructed to avoid discrimination against women; in doing so, the visibilisation of gender bias is necessary, though perhaps not sufficient. Is not ignorance that is behind the facts described by Elisa Chilet, but the exertion of male power in the gender regimes of the health systems. Relational theory is maybe the way to produce knowledge useful for eroding male-dominated gender regimes, as Connell says:

Relational theory is the approach that gives a central place to the patterned relations between women and men (and among women and among men) that constitute gender as a social structure. It explores the social practices that are shaped by, address, and modify this structure. (4)

I warmly invite Elisa Chilet to continue in this direction.
